# Compression-Responsive Photonic Crystals Based on Fluorine-Containing Polymers

**DOI:** 10.3390/polym11122114

**Published:** 2019-12-16

**Authors:** Julia Kredel, Markus Gallei

**Affiliations:** 1 Ernst-Berl Institute of Technical and Macromolecular Chemistry, Technische Universität Darmstadt, Alarich-Weiss-Straße 4, 64287 Darmstadt, Germany; j.kredel@mc.tu-darmstadt.de; 2 Chair in Polymer Chemistry, Universität des Saarlandes, Campus Saarbrücken, 66123 Saarbrücken, Germany

**Keywords:** stimuli-responsiveness, fluoropolymers, core-interlayer-shell particles, emulsion polymerization, melt-shear organization, self-assembly

## Abstract

Fluoropolymers represent a unique class of functional polymers due to their various interesting and important properties such as thermal stability, resistance toward chemicals, repellent behaviors, and their low refractive indices in comparison to other polymeric materials. Based on the latter optical property, fluoropolymers are particularly of interest for the preparation of photonic crystals for optical sensing application. Within the present study, photonic crystals were prepared based on core-interlayer-shell particles focusing on fluoropolymers. For particle assembly, the melt-shear organization technique was applied. The high order and refractive index contrast of the individual components of the colloidal crystal structure lead to remarkable reflection colors according to Bragg’s law of diffraction. Due to the special architecture of the particles, consisting of a soft core, a comparably hard interlayer, and again a soft shell, the resulting opal films were capable of changing their shape and domain sizes upon applied pressure, which was accompanied with a (reversible) change of the observed reflection colors as well. By the incorporation of adjustable amounts of UV cross-linking agents into the opal film and subsequent treatment with different UV irradiation times, stable and pressure-sensitive opal films were obtained. It is shown that the present strategy led to (i) pressure-sensitive opal films featuring reversibly switchable reflection colors and (ii) that opal films can be prepared, for which the written pattern—resulting from the compressed particles—could be fixed upon subsequent irradiation with UV light. The herein described novel fluoropolymer-containing photonic crystals, with their pressure-tunable reflection color, are promising candidates in the field of sensing devices and as potential candidates for anti-counterfeiting materials.

## 1. Introduction

In the recent past, the self-assembly of monodisperse colloidal particles attracted enormous attention due to their unique ability to crystallize into well-ordered structures [1,2,3,4]. This effect has been observed for several particle-based structures in nature, where a periodic modulation of the refractive indices within underlying microstructures can occur [5,6,7]. In this regard, synthetic colloidal crystals, such as photonic band gap materials, gained a great deal of attention as potential candidates for various optoelectronic applications [8,9,10,11,12,13]. In the pioneering work of Yablonovitch [14] and John [15], the optical features of 3D photonic band gap materials were predicted. A recent review in this field has been published by López et al. [2]. The major advantages of colloidal crystals are their low cost and convenient bottom-up fabrication, leading to remarkable optical performances with brilliant reflection colors according to Bragg´s law of diffraction [16,17,18,19,20,21,22,23,24]. There are different preparation methods for designing core-shell particles, such as coaxial electrospraying, which can be used to produce multilayer particles [25,26,27,28]. Other methods involve emulsion polymerization strategies, which is the method of choice for the present study. The fabrication of monodisperse polystyrene (PS) and poly (methyl methacrylate) (PMMA), as well as silica nanoparticles are reported for particle sizes ranging from 100 nm to 1 µm by using a variety of different polymerization techniques and the Stöber method for silica particles, respectively [8,29,30]. The majority of these techniques allows for the relatively convenient production of particles followed by self-assembling methods for the preparation of photonic crystals. However, the obtained polymeric structures usually featured only a low refractive index contrast and a lack of order on the large scale along with the presence of cracks and defects. The first issue can be overcome by the use of core particle templates for the preparation of inverse opals or by infiltration of the interstices between the template particles with high refractive index material precursors [31,32,33]. Upon the removal of the core particles templates and the solidification of the precursor material, significant differences for the refractive indices between the air voids and matrix can thus be obtained [34]. Additionally, the optical properties, i.e., the brilliance of color within opal films, can be achieved by the addition of carbon black [35,36]. In general, the addition of small amounts of carbon was found to dramatically enhance the perceived reflection color due to spectrally resonant scattering inside the opal film structure without an influence on the Bragg conditions [16,37,38].

Highly fluorinated polymers represent a unique class of functional materials, as they offer a variety of exciting properties, such as the remarkable resistance toward chemicals, their thermal stability, wetting behavior, as well as low refractive indices, compared to other polymer-based materials [39,40,41]. Therefore, the field of application is widespread and ranges over membranes, coatings, high-performance elastomers, and optical applications [42,43,44]. The design of fluoropolymers featuring a hierarchical architecture and order is relevant for the development of new applications, for example in the field of functional coatings and photonic materials. Particle-based architectures, such as colloidal crystals with adjustable dimensions have attracted increasing attention due to their potential applications in fields of catalysis, separation technologies, and optical sensors [45,46,47,48,49,50,51,52]. Monodisperse colloidal particles feature the intrinsic capability of self-assembling from their dispersion, which allows the formation of photonic crystals. Different methods for the production of opal films starting from the dispersion of monodisperse colloids have been reported [35,53,54,55]. Here, sedimentation by taking advantage of a gravitational field for monodisperse colloidal particles was one of the first techniques used to self-assemble nanospheres into a 3D-ordered lattice [56,57]. Alternatively, photonic crystals could be prepared by vertical deposition [58,59] or dip coating [60] and supported by sonication [61] or spin coating. In recent years, combinations of melting and shear ordering methods have been successfully applied for the preparation of free-standing opal films at an industrially relevant length scale [36,37,62,63]. The so-called melt-shear organization technique has the particular advantage that it completely avoids the necessity of dispersion media or organic solvents during the particle-ordering step. Moreover, fully crack-free opal films could be obtained. Recently, the feasibility of this technique for the combination of organic and inorganic core particles featuring a soft and melt-able shell has been reported [64,65,66,67]. The melt-shear organization technique allowed for the rapid fabrication of highly ordered core particles embedded in a shell of elastomeric polymer matrix [37,68]. By the incorporation of functional polymers and the feasibility of different external triggers, the lattice spacing inside the opal film can be varied on demand by taking advantage of the stimuli responsiveness of the used polymers. The switching capabilities are accompanied with a reversible change of the reflected color [69,70,71,72]. Recent progress in the field of tunable colloidal crystals of monodisperse polymer-grafted core-shell particles, embedded in a responsive polymer matrix, lead to easily tunable materials, which are interesting candidates for sensing devices [13]. Stimuli-responsive opal films have been previously described for sensors by the application of external triggers, for instance, temperature [73,74], electric and magnetic fields [75,76], pH variations [77,78], humidity [79], ionic strength [80], organic solvents [81,82], and mechanical stress. Especially mechanically responsive materials have attracted a lot of attention in the last years [83,84,85,86,87].

Within the present study, the first combination of soft core and soft shell particles only featuring an adjustable hard interlayer for the preparation of compression-responsive photonic crystals is reported. We investigate how the refractive index contrast between the core and the shell material can be increased by combining highly fluorinated shell materials and aromatic copolymers as core materials. Based on the core-interlayer-shell architecture and the presence of well-defined spherical particles, opal films can be produced by the application of the melt-shear organization technique, which involves processing with moderate pressure and temperature. By the incorporation of UV cross-linking agents and investigating the UV cross-linking capabilities, the prepared patterns inside the opal films can be fine-tuned or fixed after the application of spatially restricted pressure, which locally changes the reflection color of the opal film.

It is expected that the resulting novel opal films are interesting candidates for compression-responsive (reversible) sensing materials and potential candidates for anti-counterfeiting materials, based on their convenient UV-mediated cross-linking chemistry, reversibly switchable reflecting colors, and the fixation of the written patterns.

## 2. Experimental Section

### 2.1. Reagents

Benzyl acrylate (BzA, 98%), allyl methacrylate (ALMA, 98%), styrene (S, 99%), ethyl acrylate (EA, 99.5%), and sodium dodecylsulfate (SDS) were purchased from Fisher Scientific (Schwerte, Germany). Potassium hydroxide flakes (KOH), sodium peroxodisulfate (SPS), and sodium metabisulfite (SBS) were purchased from Sigma-Aldrich (St. Louis, MO, USA), benzophenone was purchased from Merck Chemicals (Darmstadt, Germany), Irgacure 184 and butandiol diacrylate (BDDA) were purchased from BASF (Ludwigshafen, Germany), carbon black (Special Black 4) was purchased from Degussa GmbH (Essen, Germany), and Dowfax 2A1 were obtained from Dow Chemicals (Midland, MI, USA). The chemicals were used as received, if not otherwise mentioned. Prior to the polymerization, the inhibitor was removed from all monomers by passing through an alumina column (basic, 50–200 µm, Acros Organics, Thermo Fisher Scientific, Schwerte, Germany). The fluorinated monomer, 2-((1, 1, 2-trifluoro-2-(perfluoropropoxy) ethyl) thio) ethylacrylate (entitled fluoroacrylate in the following) was obtained from Merck KGaA (Darmstadt, Germany) and distilled under reduced pressure.

### 2.2. Instrumentation

Transmissions electron microscopy (TEM) was accomplished on a Zeiss EM10 (Oberkochen, Germany) with an operating voltage of 60 kV. The images were recorded with a slow-scan CCD camera TRS (Tröndle) in bright field mode. The control of the camera was computer-assisted using Image SP from TRS. To measure the particle diameter, at least 50 particles were measured with respect to their size with the program Image SP Viewer. For these investigations of the particles, the diluted particle dispersion was drop-cast on a copper grid (Plano GmbH, Wetzlar, Germany) and dried overnight at room temperature. The copper grids were coated with a nitrocellulose film prior to casting the particle dispersion. Dynamic light scattering (DLS) measurements of the particles were performed on a Zetasizer ZS90 (Malvern Instruments, Malvern, UK). The experiments were carried out for the diluted particle dispersion, after every step of polymerization, at an angle of 90° and at a temperature of 25 °C. For particle diameter evaluation, the z-weight average of the hydrodynamic diameter was used. For determining the thermal properties of the synthesized particles, differential scanning calorimetry (DSC) was performed with a Mettler Toledo DSC-1 (Columbus, Ohio, USA) in a temperature range of −100 to 200 °C with a heating rate of 20 K·min^−1^ under nitrogen atmosphere. For scanning electron microscopy (SEM), a Philips XL 30FEG (Philips, Amsterdam, Netherlands) with an operating voltage of 15 kV was used. The samples’ surface was cleaned with a Femto plasma cleaner (Diener electronic GmbH, Ebhausen, Germany) and followed by coating with a thin layer of Pd/Pt (8 nm), using a Quorum Q300T D sputter coater (Lewes, UK). Angle-dependent reflection UV-Vis measurements were performed using a custom-made goniometer setup enabling measurements in steps of 10° with scattering angles between 90° and 50°. All other reflection spectra were recorded with an angle of incident light of 90° using a Vis/-NIR fibre spectrometer USB 2000 from Ocean Optics (Ostfildern, Germany). For these measurements, a deuterium/tungsten halogen lamp DT mini 2 from Ocean Optics was used. For extrusion of the polymer together with the other components (carbon black, UV cross-linking reagents), a microextruder HAAKE Minilab II350 (Thermos Scientific, Schwerte, Germany) was used with a rotation speed of 120 UPM at a temperature of 50 °C. For opal film formation via melt shearing, the polymer mass was covered by two PET foils and heated between two steel plates inside a Colin laboratory press (Dr. Colin GmbH, Ebersberg, Germany). The opal films were irradiated with a mercury lamb of a UVA-Cube 2000 (Dr. Hoenle AG, Gräfelfing, Germany) with an output power of 1000 Watt at a distance of 10 cm, as given within the main text. To quantify the pressure on the finished opal films, they were treated with the same Colin laboratory press (Dr. Colin GmbH, Ebersberg, Germany). The color change can be followed by applying a pressure of 2 bar in both cases. However, a slight color change of the opal films can be observed even at a pressure between two fingers. To determine the refractive index of the poly (fluoro-acrylate), ellipsometry measurements were carried out with a polarizer-compensator-sample analyzer (PCSA) ellipsometer (Optrel GbR, Sinzing, Germany) at a wavelength of 632.8 nm. The measurements were performed at an angle of incidence of 70° under ambient conditions (room temperature and 30% rh). The data were fitted with Elli v3.1 (Optrel GbR, Sinzing, Germany) based on the following two-layer model for the polymer shown in Table 1.

### 2.3. Synthesis of Core-Interlayer-Shell Particle via Emulsion Polymerization

**Figure 1:**
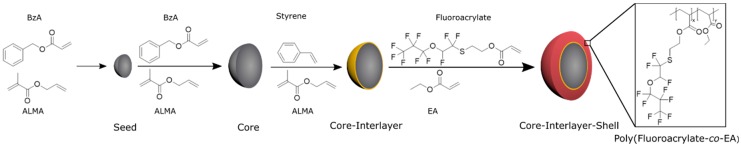
Stepwise synthesis of core-interlayer-shell particles. The seed and core particles consist of poly (benzyl acrylate-*co*-allyl methacrylate) (BzA and ALMA), the interlayer contains styrene and allyl methacrylate (ALMA) and the shell material is formed by 2-((1, 1, 2-trifluoro-2-(perfluoropropoxy) ethyl) thio) ethylacrylate (fluoro-acrylate) and ethyl acrylate (EA).

The stepwise fabrication of core-interlayer-shell particles is illustrated in Figure 1 and was accomplished according to starved-feed emulsion polymerization protocols, as given in the following. Under nitrogen atmosphere, a 250 mL double-wall reactor equipped with a reflux condenser and stirrer is heated up to 80 °C and filled with a cold monomer emulsion consisting of 93 g of deionized and degassed water, 0.01 g of SDS, 1.3 g of BzA, and 0.013 g (10 wt %) of ALMA. The monomer emulsion was stirred with a velocity of 350 UPM. In this sequence, the polymerization is immediately initiated by adding 16 mg of sodium metabisulfite (SBS), 150 mg of sodium peroxodisulfate (SPS), and 16 mg of SBS solved in 5 mL of deionized water. After 10 min, a monomer emulsion containing 37.2 g of BzA, 3.7 g of ALMA (10 wt %), 0.1 g of KOH-flakes, 0.13 g of SDS, 0.13 g of Dowfax 2A1, and 46.5 g of water were continuously added with a rotary piston pump and a flow rate of 0.4 mL/min, within 4 h. Prior to dosing, the monomer mixture was treated with ultrasonic to generate a stable emulsion. After 1 h of reaction time, the poly (BzA-*co*-ALMA) particles featuring an average hydrodynamic diameter of 217 nm and a solid content of 14.3 wt % were characterized via DLS and TEM measurements and stored for further use.

The core-interlayer-shell particles, featuring a rigid interlayer and a soft shell material, were synthesized in a 100 mL double-wall reactor, which was equipped with a stirrer and a reflux condenser under nitrogen atmosphere at 80 °C and a velocity of the stirrer of 350 UPM. For the emulsion polymerization, 31.3 g of the core emulsion of poly (BzA-*co*-ALMA) and 20 g of water was filled into the reactor, and the polymerization was started by adding 6 mg of SBS, 69 mg of SPS, and 6 g of SBS dissolved in 2 mL of deionized water. After 10 min, a monomer emulsion (ME1) consisting of 0.75 g of styrene, 0.25 g of ALMA (25 wt % of monomer), 10 mg of SDS, 20 mg of Dowfax 2A1, and 6 g of water were continuously added with a dosing rate of 0.2 mL/min (Interlayer 1). After the complete addition of ME1, a sample of the core-interlayer particle dispersion was taken for characterization, and a solution of 11 mg of SPS in 2 mL of water was added to start the polymerization of the shell material. For this purpose, a monomer emulsion containing 4.9 g of fluoro-containing acrylate, 0.45 g of EA, 7 mg of KOH, 16 mg of SDS, 13 mg of Dowfax 2A1, and 10 g of water were added with a dosing rate of 0.2 mL/min (shell 1). In the next step, the temperature of the polymerization mixture was maintained for one additional hour prior to cooling to room temperature. The particle size of the core-interlayer 1-shell 1 particles were determined by DLS and TEM. The particles of type 1 for the reversible pressure-sensitive Opal Film 1 featured a core-interlayer-shell composition of 45 wt % to 10 wt % to 45 wt % of the respective copolymers.

For the synthesis of the particles type 2, the composition of the core-interlayer-shell particles was 45 wt % to 15 wt % to 40 wt %, which is slightly different as the ME1 emulsion consisted of 1.13 g of styrene, 0.38 g of ALMA (25 wt %), 15 mg of SDS, 30 mg of Dowfax 2A1, and 6 g of water. ME2 contained 3.6 g of fluoroacrylate, 0.40 g of EA, 6 mg of KOH, 14 mg of SDS, 11 mg of Dowfax 2A1, and 10 g of water.

### 2.4. Particle Processing via Melt-Shear Organisation

For the preparation of elastomeric opal films, the respective as synthesized core-interlayer-shell particles were precipitated in methanol containing approximately 30 wt % of saturated sodium chloride solution, followed by filtration and washing with methanol and deionized water, and the particles were finally dried overnight under reduced pressure at room temperature. Then, 4 g of the elastomeric polymer mass (particle type 1) was mixed together with 0.4 g (10 wt %) of BDDA, 25 mg of benzophenone, 25 mg of Irgacure 184, and a portion of 0.5 mg carbon black in a microextruder at 50 °C and 120 UPM rotation speed to produce the corresponding extrusion strands. The particles type 2 were obtained by using the same protocol, but after mixing with 0.6 g (15 wt %) of BDDA, 25 mg of benzophenone, 25 mg of Irgacure 184, and 0.5 mg of carbon black. An amount of approximately 2 g of the polymer extrusion strands were covered between two PET foils (Mylar°A°75, DuPont) and two steel plates and heated in a Collin laboratory press up to 50 °C and pressed at 5 bar for 60 s. The particle mass was transduced into an opal disc film of ca. 4.5 cm in diameter. Subsequently, Opal Film 1 was irradiated with a mercury lamb of a UVA cube for 5 min from both sides of the opal film. Afterwards, the PET foils was removed, and the opal disc was investigated with respect to its reversible pressure-sensitive properties. To produce the irreversibly pressure-sensitive opal film (Opal Film 2), the film was first pre-cross-linked inside the UV cube for 1 min. A pattern was pressed inside the film disc followed by additional cross-linking for 5 min using the UV cube for each side of the film.

## 3. Results and Discussion

A promising technique for the preparation of highly ordered opal films featuring brilliant reflection colors is the so-called melt-shear organization technique [53]. For the application of this technique, core-shell particles were used consisting of a highly cross-linked—and compared to the shell material—hard core material and a soft polymeric shell material [88]. The following chapter is divided into five sections, starting with the preparation and characterization of the herein investigated core-interlayer-shell particles, followed by the preparation of the opal film via the melt-shear organization, the investigation of the optical properties, and finally the investigation of the compression-responsive behavior of the opal films.

### 3.1. Synthesis and Characterization of Core-Interlayer-Shell Particles

In contrast to the reported core-shell particles consisting of a hard core and a soft polymeric shell material [89], different particle systems were prepared: here, the combination of soft core-soft shell material will be prepared, but consisting of a comparably hard interlayer between the core and the shell will be produced. For this purpose, the core-interlayer-shell particles were synthesized by using a semi-continuous stepwise emulsion polymerization (Figure 1). In the first step, poly (benzyl acrylate-*co*-allyl methacrylate) seed particles were synthesized in a batch process, which was initiated by using a redox initiator, followed by the continuous addition of benzyl acrylate (BzA) and allyl methacrylate (ALMA) in order to prepare the core particles in the second step. The continuous addition of a monomer leads to almost perfect particles, which feature allylic cross-linking moieties by incorporation of the ALMA monomer. Although the particles could be considered as cross-linked gels, these soft particles maintain their shape during synthesis and characterization. In the subsequent step, a thin but comparably hard interlayer consisting of poly (styrene-*co*-allyl methacrylate) was prepared by emulsion polymerization strategies. Finally, in order to obtain a soft processable shell material featuring a lower refractive index contrast (compared to the core material), 2-((1, 1, 2-trifluoro-2-(perfluoropropoxy) ethyl) thio) ethylacrylate (fluoro-acrylate) and ethyl acrylate (EA) was partially grafted onto the cross-linking agent in the interlayer based on the radical polymerization with the allylic moieties of the interlayer. Due to its low glass transition temperature (−25 to −30 °C) but immobilization to the hard interlayer, the soft poly (fluoro-acrylate-*co*-EA) shell was capable of embedding the core particles into a colloid crystal structure inside an elastomeric matrix material after melt shearing (cf. following section).

**Figure 2:**
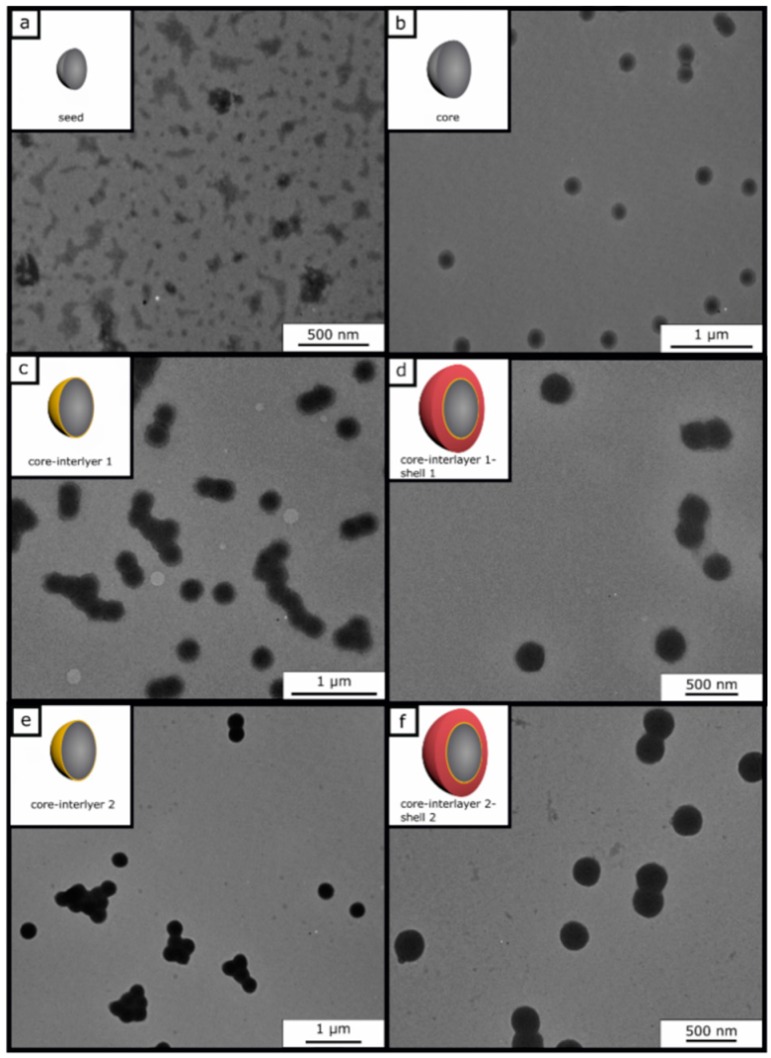
TEM measurements of the two different particle batches for herein described investigations: seed particles (**a**); core particles (**b**); core-interlayer 1 particles (**c**); core-interlayer 1-shell 1 particles (**d**); core-interlayer 2 particles (**e**) and core-interlayer 2-shell 2 particles (**f**). For both types of core-interlayer-shell particles, the same core was used.

These core-interlayer-shell particles were synthesized with two different thicknesses for the interlayer to generate different opal films for reversible (Opal Film 1) and irreversible (Opal Film 2) pressure-sensitive opal films. For the synthesis of different core-interlayer-shell particles, the same core materials was used, while different amounts of monomers for the interlayer were grafted to the core material, i.e., either 10 wt % (interlayer 1) or 15 wt % (interlayer 2). These different amounts—given as weight percentage—of the poly(styrene-*co*-allyl methacrylate) particle batches were prepared to study the effect of the thickness of the interlayer upon compression of the final opal films. Furthermore, the amount of the monomers for the shell (45 wt % for shell 1 and 40 wt % for shell 2) was varied in both cases. To prove the success of the stepwise preparation of the core-interlayer-shell particles with respect to their morphology and average size, dynamic light scattering (DLS) measurements and transmission electron microscopy (TEM) measurements were carried out. TEM images of each step of the emulsion polymerization for both types of core-interlayer-shell particles with different recipes and thicknesses of interlayers and shells are given in Figure 2. From these TEM images, it can be concluded that well-defined particles were obtained comprising seed, core, core-interlayer, and core-interlayer-shell particles. The average sizes for all particles, as obtained from TEM and DLS measurements, are compiled in Table 2.

**Figure 3:**
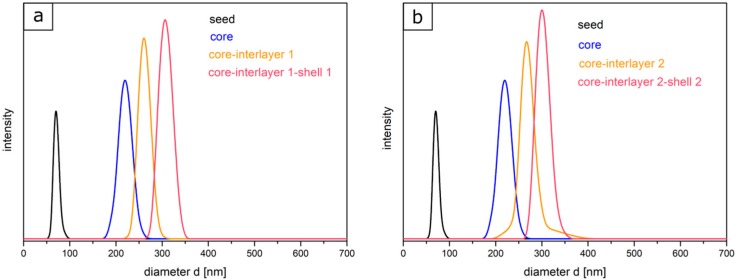
DLS measurements for the different particle dispersions in water for the two particle batches type 1 (**a**) and 2 (**b**).

Figure 2 shows the TEM images of each particle synthesis step. The seed particles of poly (BzA-*co*-ALMA) (a) demonstrated no well-defined particle size distribution of individual particles, which is typical for the batch process in emulsion polymerization. Moreover, the very soft polymer material, the small particle sizes, and agglomeration of the soft particle mass during sample preparation are explanations for issues with seed particle investigations. Upon drying, the soft particle mass merges together, leading to an ill-defined polymer mass during sample preparation and characterization using TEM. This effect is typically observed for very soft seed particles, as obtained by emulsion polymerization strategies. On the other side, the core particles are more separated and therefore feature a well-defined spherical shape and size. Moreover, the polymerization of the interlayer and shell could be proven by the increasing size of the particles, as derived by TEM measurements (Table 2). In general, the core, core-interlayer, and core-interlayer-shell particles feature a uniform spherical shape, evidencing the subsequent growth at every step of emulsion polymerization. For the determination of the average particles sizes out of the TEM images, 50 particles were measured. The sizes of the dried particles were obtained from the TEM studies and are compiled in Table 2 along with their standard deviations. For comparison, DLS measurements were performed, which generally measure the hydrodynamic diameter of the particles (Figure 3).

In comparison to TEM investigations, the size distributions and the hydrodynamic diameters of particles of each step were characterized by using DLS measurements. It is worthy to mention that the particle diameter—obtained via DLS—is generally significantly larger than the diameter measured with TEM, since here, the hydrodynamic volume in the swollen state of the particles is determined, which is larger compared to the particles in the dried state. Starting with the seed particles over the core particles, core-interlayer particles, and finally the core-interlayer-shell particles, a continuous increase of the average particle diameter could be observed. Moreover, a narrow size distribution with a small standard deviation (<5%) confirmed the successful synthesis of well-defined particles in both cases, which is essential for the fabrication of colloidal crystals, as described in the following section.

To summarize, the obtained core-interlayer-shell particles allows for the subsequent preparation of opal films with brilliant reflection color via the melt shear organization technique, which will be described in the following section.

### 3.2. Preparation of Opal Films via Melt-Shear Organisation Technique

**Figure 4:**
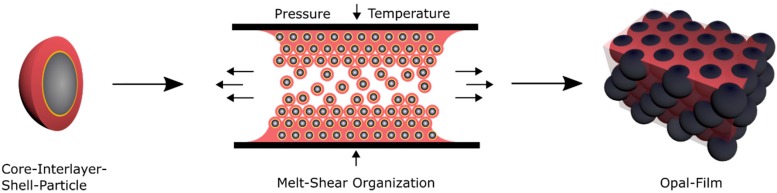
Fabrication of opal films using the melt-shear organization technique.

As mentioned within the introduction, a promising technique for the preparation of elastomeric opal films based on core-shell particles was developed in the last years [62,63,90]. This technique enables the formation of easy-scalable and free-standing opal films with distinct reflection colors as a function of incident angle according to Bragg´s law of diffraction [91]. In the current work, opal films were prepared and investigated that do not change their color due to their lattice plane distance upon changing the amount of matrix material, as described in previous studies [16,92,93]. Here, the soft core particles are considered to enable the deformation of the spherical particle shape by moderate pressure in a reversible or an irreversible manner. For investigation of these features, different prerequisites for the tailored particle architecture in order to produce opal films via the melt-shear organization technique are necessary. First, the soft elastomeric shell material has to be processable under ambient temperatures—similar to previously reported elastomeric opal films—without core particle deformation. Furthermore, in order to enable the self-organization of the core particles inside the matrix, the shell must be grafted to the cores, to ensure leaving the soft polymer mass from the interlayer-core particles, during extrusion or during the melt-shear organization. The most important aspect for the herein investigated soft core-hard interlayer-soft shell particle architecture is that moderate applied pressure does not change the original particle shape. Therefore, the crucial step within the present study is the protection of the soft core material against deformation and shell detachment by generating a stable interlayer, i.e., by sufficient interlayer thickness and/or cross-linking sites. During the melt-shear organization, the polymer mass is placed between two plates of an industrial press. While increasing the pressure and temperature, the core-interlayer particles are capable of merging into the colloidal crystal structure. In general, the elevated temperature leads to a softening of the polymer shell, which is accompanied with a decreasing viscosity of the particle mass. Through increasing the pressure, the core particles inside the matrix can be hexagonally arranged, as schematically depicted in Figure 4.

**Figure 5:**
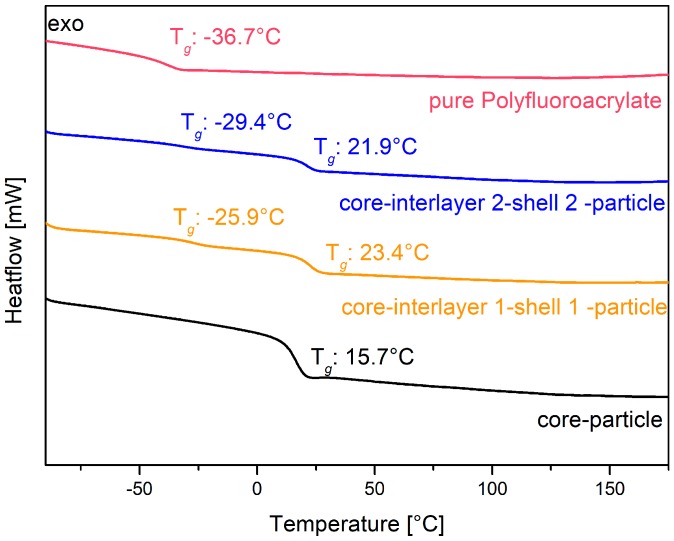
Differential scanning calorimetry (DSC) measurements of core material, both types of core-interlayer-shell particles and of the pure poly (fluoro-acrylate) of the shell material.

The core particles consisting of poly (BzA-*co*-ALMA) were used for the preparation of the pressure responsive opal films, and they revealed a glass transition temperature of only 15.7 °C, as determined via DSC measurements (see Figure 5, black line). This temperature is slightly higher than the glass transition temperature of about 6 °C for pure poly(benzyl acrylate) [94]. The higher glass transition temperature is due to the content of the respective co-monomer ALMA, which stabilized the core particles by cross-linking reaction. The interlayer of the particles enabled the particle architecture to keep their shape and for the further introduction of an outer polymer shell. This thin layer featuring a slightly higher glass transition temperature enabled the melt-shear organization process without deformation of the soft particle cores, as described in the following section. The balance with respect to the size and glass transition of the interlayer shell was of crucial importance, as it should avoid core particle deformation during processing, but at the same time, a deformation of the interlayer and also the core should be possible by an appropriate application of (desired) external pressure. Therefore, the interlayer has to be adjusted with a thickness of 17 nm for interlayer 1 and 26 nm for interlayer 2. In order to achieve reversible pressure sensitivity, it was essential that the intermediate shell was not detached by the applied pressure, but only deformed. This can be accomplished by using poly (styrene-*co*-ALMA) as a cross-linked interlayer. In more detail, the glass transition temperature of this interlayer material was found to be comparable according to the literature in a range of 100 to 105 °C [95]. Therefore, the processing temperature during the melt-shear organization must not exceed 100 °C. The cross-linker ALMA fulfilled two functions within the particle architecture: (i) stabilization of the interlayer by cross-linking reactions and (ii) enabling subsequent grafting of the—again—soft shell to the core-interlayer particles. Here, the outer shell of the core interlayer shell particle consisted of the copolymer of poly (fluoro-acrylate-*co*-EA). In this particular case, the poly (fluoro-acrylate)-containing copolymer was used because it is comparably soft and featured a rather low refractive index. The glass transition temperature of the soft fluorine-containing shell material was determined to be in the range of −25 to −29 °C, as obtained by DSC measurements. The corresponding thermograms are given in Figure 5 as a yellow graph for particles 1 and as a blue line for particles 2. The obtained value was found to be between the glass transition temperature of the corresponding pure polymers (see Figure 5: −36.7 °C for the homopolymer poly (fluoro-acrylate) (red line) and −24 °C for PEA [96]). The second observed glass transition temperature was determined to stem from the core particle material consisting of poly (benzylacrylate-*co*-ALMA), as described above. The use of this soft shell material allowed for a convenient processing at low temperatures, so that the cores maintained their spherical shape. After the stepwise production of the particles by using emulsion polymerization protocols, the polymers were precipitated and dried at low temperatures in vacuum for the intended production of the opal films.

In the next step, the particle mass was processed via extrusion and melt shearing, and it was used for UV-mediated cross-linking reactions. For this purpose, the dried particles were mixed in an extruder with a system of different photo-cross-linking agents, prior to use in the melt-shear organization process (cf. Experimental Section). The UV cross-linkers served to generate stable and elastic photonic crystals after melt shearing of the particles. An efficient strategy for subsequent UV-mediated opal film cross-linking has been reported by Viel et al. [92]. As in the present study, the polymer shell did not feature residual acrylic functionalities to ensure an efficient UV cross-linking reaction; additional 1, 4-butandiol diacrylate (BDDA) was used as a cross-linking monomer in addition to a UV initiator system (benzophenone and Irgacure 184, cf. Experimental Section). The cross-linking reaction was performed after opal film formation via proton abstraction of the polymer chains and subsequent radical recombination [92,93]. After incorporation of the cross-linking components in the polymer composition, the polymer-extrusion strands were placed between the plates of a press and upon application of moderate temperatures (50 °C) and pressure (5 bar). Compared to previous studies, the pressure was relatively low, as the core particles should not be deformed. The influence of the amount of BDDA and photoinitiator was varied by using two different compositions. These opal films derived from the herein investigated core-interlayer-shell architectures and cross-linking components featured elastomeric properties exhibiting reversible and irreversible color changes, which will be characterized in more detail in the following section.

### 3.3. Optical Properties and Morphology of the Core-Interlayer-Shell-Based Opal Films

The specific requirements for the thermal and mechanical properties of the core-interlayer-shell particle systems for the opal films and the optical properties of the herein investigated particle-based films will be investigated in this section. First, the optical properties of the opal film must fulfill the condition for structural colors based on spherical particles.

(1) $$\lambda_{111}=2\alpha_{111}{\left(n_{eff}^{2}-\sin^{2}\theta \right)}^{1/2}$$

According to Bragg´s law of diffraction, and in combination with Snell´s law (Equation (1)), the color of reflected light depends on the angle of incident light (θ) and on the lattice plane distance $\left(\alpha_{111}\right)$, which is influenced by the diameter of the particles. Another important parameter is the effective refractive index (n*_eff_*), which can be calculated from the volume fractions ($\phi_{i})$ and the refractive indices (n*_i_*) of the individual components according to Equation (2).

(2) $$n_{eff}=\sum^{\;}n_{i}\phi_{i}$$

The value for the herein investigated opal film resulted from the core particles, featuring a value for the refractive index of poly(benzyl acrylate) (*n* = 1.55) [97] from the interlayer with polystyrene (*n* = 1.58) [98] and the shell material consisting of poly(ethyl acrylate) (*n* = 1.47) [63] and poly (fluoro acrylate) (*n* = 1.39). The latter value was measured by using an ellipsometer at a wavelength of 632.8 nm for a pure polymer film featuring a thickness of 80 nm. The values were fitted with a two-layer model (cf. Experimental Section). Compared to previously reported core-shell particle opal films [52], the combination of these components with poly (BzA), with a high refractive index and the fluoropolymer featuring a low refractive index, a sufficient refractive index contrast of (Δ*n_eff_* = 0.19) could be obtained, which should also lead to structural colors with good optical properties. It is worthy to mention that previous studies on elastomeric opal films typically feature an efficient refractive index contrast of Δ*n_eff_* = 0.12 [99].

**Figure 6:**
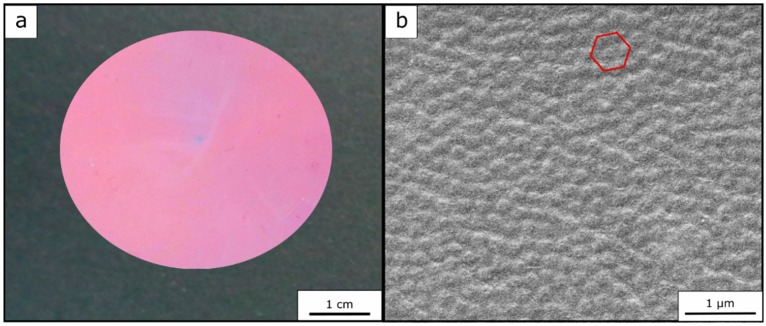
Photography of Opal Film 1 (**a**) and corresponding SEM topography image (**b**) of the surface of the same opal showing the underlying core particles inside a matrix.

In addition to these requirements for fulfilling the conditions of structural colors, there must be also a sufficient order of the particles inside the matrix material, so that a high periodicity and refractive index modulation becomes possible. The particle order was examined by means of scanning electron microscopy (SEM) for the top surface of herein prepared opal films (see Figure 6b). Furthermore, a photography of the opal film is shown in Figure 6a, again proving the fulfilled requirements of a brilliant—in this case red—reflection color.

In Equation (1), the dependency of the reflected color on the angle of incident light is described. To determine the Bragg peak at different angles of incidence of light and to prove the good optical properties of the opal films, angle-dependent UV/-Vis measurements were carried out.

**Figure 7:**
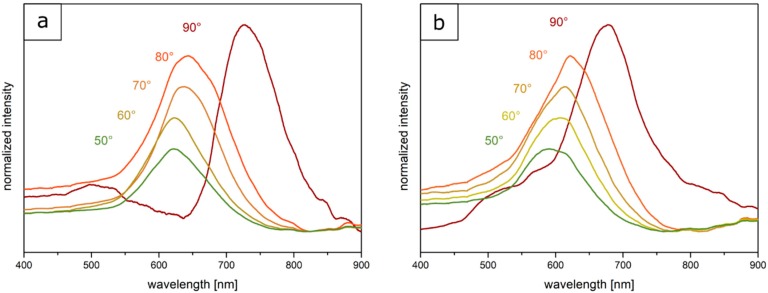
Angle-dependent UV-Vis reflection spectra of the Opal Film 1 (**a**) and Opal Film 2 (**b**) prepared from soft core-rigid interlayer-soft shell particles.

For this purpose, the measurements were performed at angles of incident light between 90° and 50°. It can be concluded from the corresponding spectra (Figure 7a) that the optically red Opal Film 1 featured a reflectance peak according to Bragg´s law of diffraction at 90° at a wavelength of 726 nm. At smaller angles of incidence, the Bragg peak shifted from 726 nm at 90° to 619 nm at 50° into the green regime. These findings prove the existence of a structural color of the photonic crystals. Figure 7b shows the angle-dependent UV/-Vis measurements of the Opal Film 2. The reflection peak shifted from 678 nm at 90° to 589 nm at 50°, once again evidencing the presence of a structural color for the fluoropolymer-containing opal films.

In conclusion to the investigated optical properties, the successful synthesis of the tailored particles featuring a soft core, rigid interlayer, and soft shell was demonstrated, fulfilling all requirements for the creation of brilliant opal films.

### 3.4. Reversibility and Pressure-Responsiveness of Investigated Opal Films

**Figure 8:**
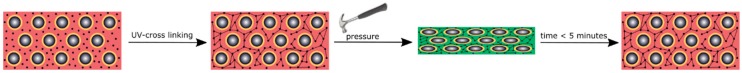
Schematically presentation of the cross-linking reaction for the preparation of pressure-sensitive opal films.

The elastomeric Opal Film 1 was prepared in order to apply a moderate pressure for an observable color change. Moreover, based on the particle architecture and cross-linking reaction capabilities, the optical properties should be reversible after removal of the moderate pressure between two fingers to reversibly return to the original reflection color upon relaxation. For this purpose, 10% of the cross-linking monomer BDDA and the UV initiators benzophenone and Irgacure 184 were incorporated by extrusion into the polymer particle mass prior to the processing, i.e., extrusion and melt shearing. Furthermore, the addition of the liquid monomer BDDA led to the formation of extrusion polymer strands that were soft, sticky, and easy to process within the melt-shear organization. The opal film was obtained between two PET foils and two press plates at 50 °C and five bar for 60 s. These are comparatively mild conditions for the melt-shear organization process, as described previously [65]. The opal film was cooled to room temperature followed by subsequent cross-linking reaction by irradiation using a UVA cube (cf. Experimental Section). As the irradiation cannot completely interfuse the film with a thickness of approximately 500 µm, the opal film was irradiated from both sides for 5 min. Cross-linking of the opal films has been shown to be feasible for achieving satisfactory elasticity, hardness, and durability [16,89,92,99]. In the absence of cross-linking reactions, the opal films tend to flow similar to a viscous liquid under load or stress. In Figure 8, the cross-linking and the application of mechanical stress is schematically depicted.

**Figure 9:**
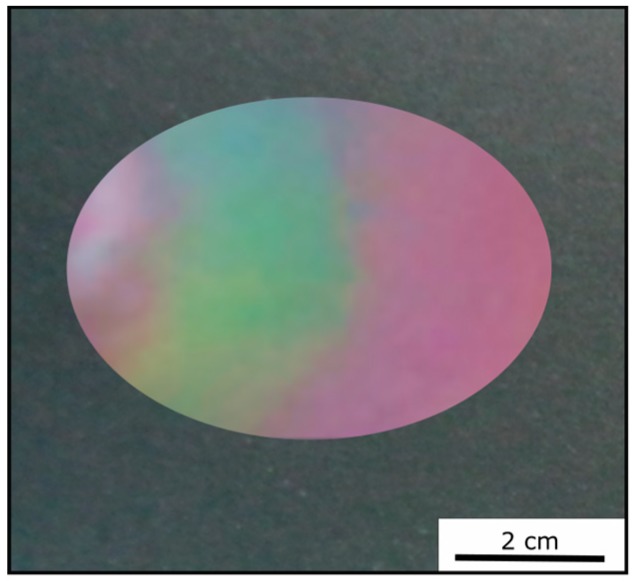
Photography of Opal Film 1 after application of a pressure of approximately 2 bar (left side).

The cross-linked Opal Film 1 offered good optical properties (see Section 3.3, Figure 6) and featured a red reflection color (Figure 7). By applying moderate pressure of approximately 2 bar for 5 s between two steel plates in a Collin laboratory press, the film changes its color and appeared green at the respective pressed area. Figure 9 exemplarily shows photography of the Opal Film 1, which was compressed at the left side.

**Figure 10:**
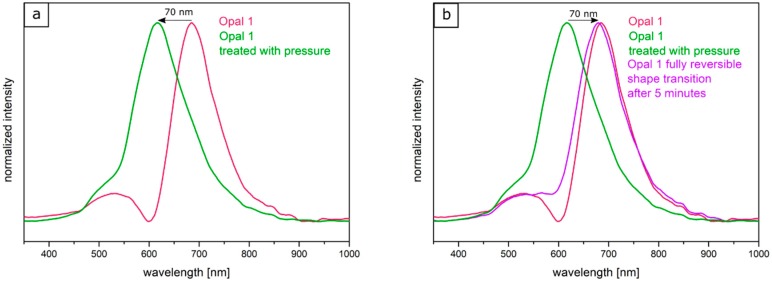
Original UV/Vis spectrum of Opal Film 1. The reflection peak is shifted through pressure by 70 nm into the green reflection regime (**a**); UV-Vis spectrum of the repeatedly compressed and relaxed opal film (**b**).

Two factors were considered for influencing the pristine optical properties of the opal films: (i) the entire opal film compression leading to a change of the spherical particle to particle distances, and (ii) the compression of the soft core particles accompanied with deformation. Both factors lead to a blue-shift of the color. The corresponding particle interlayer was considered not to withstand the applied pressure of 2 bar and was therefore changed upon pressure. Since the interlayer was not brittle, only a deformation of the soft-core particles took place. Due to the reduced lattice space between the core particles, the wavelength of the reflected light changed according to Equation (1), and the film appeared green. This change of color could be measured and analyzed by UV-Vis spectroscopy measurements. From Figure 10a, it can be concluded that the reflection peak shifted by 70 nm into the green regime of the spectrum because of the applied pressure. After removal of the pressure, the film underwent a change of color again to the original state, which occurred on a timescale of a few minutes. Measuring the optical properties via UV/Vis spectroscopy again after a relaxation of 5 min lead to a red reflection color located at 680 nm of the reflective wavelength at an angle of view of 90°, which matched the Bragg peak position for the original opal film. By UV-mediated cross-linking reaction of the matrix, the opal film featured a restoring force and the optical properties were recovered to its original values, in which it was previously cross-linked. The interlayer also returned to its original spherical shape, again increasing the lattice plane distance and shifting the color of the reflected light back to the red regime of the spectrum. From Figure 10b, it can be concluded that after the removal of pressure, the reflection peak completely shifted back and featured the previous reflection color at 680 nm. This reversible color change was carried out three times for the same opal film, which showed the ability to act as a fully reversible optical compression sensor.

### 3.5. Reversible and Irreversible Shape Transition of Opal Films

**Figure 11:**
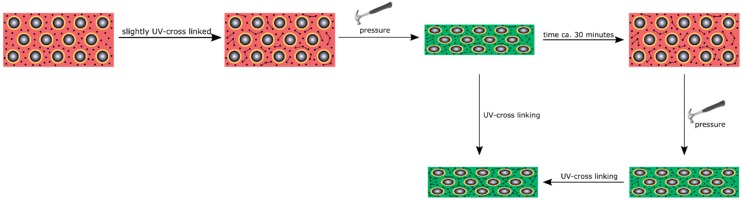
Schematic presentation of the cross-linking strategy for a reversible pressure-sensitive opal film and after a second cross-linking step, leading to irreversible shape transformation of the opal film upon fixation of the pattern.

To produce an opal film that reacts to an applied pressure both in a reversible and irreversible manner, particles were produced, as analogously described in Section 3.4, but featuring a thicker interlayer. Moreover, an UV initiator and a cross-linking monomer, BDDA, were incorporated into the polymer mass prior to the opal film formation. Herein, with 15 wt % of the particle as the polymer interlayer, a slightly thicker layer was obtained featuring a size of 26 nm. These two differences between Opal Film 1 and Opal Film 2 led to Opal Film 2 described in this section, which was still sensitive upon application of pressure (2 bar), but relaxation was prolonged now (from 5 to 30 min). This feature nicely reflected that convenient particle modification was shown to be capable of changing the entire optical properties and relaxation behavior of the opal film. Moreover, induced UV cross-linking reaction was also shown to fix a pattern, which was produced by locally restricted pressure and UV-Vis treatment. The simplified strategy is given in Figure 11.

**Figure 12:**
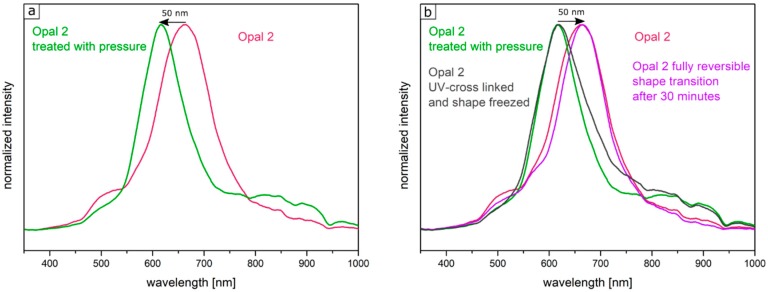
UV/Vis spectrum of Opal Film 2. The reflection peak shifted upon pressure by 50 nm into the green regime of the spectrum (**a**); Fully reversible shift of the reflection peak back to the original wavelength and corresponding spectrum after the same pressure again followed by freezing upon application of UV irradiation for 5 min from both sides of the opal film (**b**).

In order to produce a reversible relaxation of the opal film, it was mixed with UV cross-linker agents and slightly pre-cross-linked for one minute. By this, the opal film was somewhat stabilized and featured already elastomeric properties. When a pressure was applied, the soft-core particles were deformed and the lattice space was reduced, causing the film to appear in a green reflection color. After removing the pressure, relaxation took again a few minutes for the film to return to its original shape and reflection color. As this film was only slightly cross-linked, this decrease of the pressure-induced color change mainly stemmed from a higher stability of the thicker interlayer and less from the restoring force of the cross-linked matrix. Therefore, this decrease was much slower compared to the opal film described in Section 3.4. The time for full relaxation was determined to be 30 min. In order to achieve an irreversible color change based on an applied pressure, the opal film had to be significantly cross-linked after application of the pressure. For this purpose, the film was irradiated with UV light for 5 min from each side. By the high amount of incorporated UV initiator and the high amount of cross-linkable monomer in the matrix, the film was sufficiently cross-linked in its compressed form. As a result, the written pattern was frozen. Figure 12 shows the UV-Vis spectra of the corresponding Opal Film 2. Figure 12a demonstrated the shift of the reflection peak under film compression. The Bragg peak maximum was located at a wavelength of 660 nm for the untreated opal film and at a wavelength of 610 nm for the compressed opal film. Figure 12b shows the fully reversible pressure sensitivity after 30 min in the purple graph. Compared to this, the gray spectrum shows the reflection peak of the film, which was compressed in the Colin laboratory press with a pressure of 2 bar once more followed by UV-mediated cross-linking reaction for 5 min from both sides of the opal film. Here, the obtained reflection peaks did not change after measuring the opal film again after 7 days.

**Figure 13:**
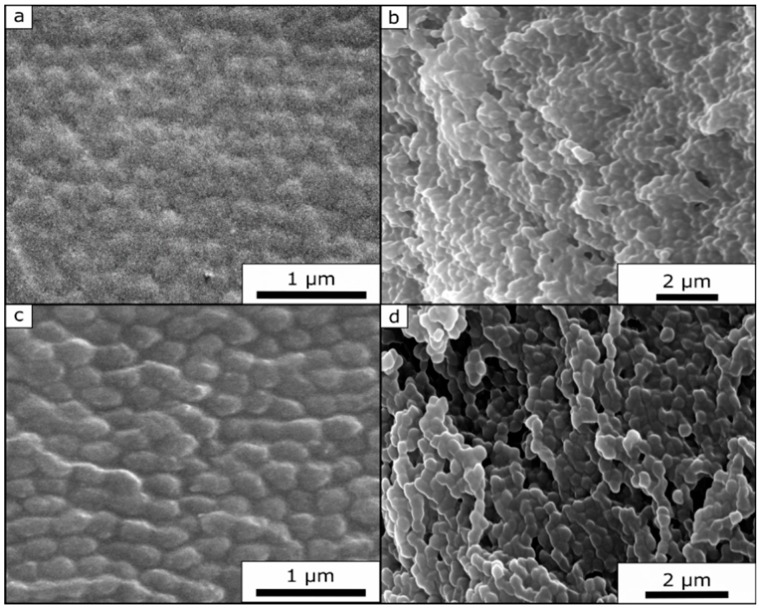
SEM Images of the untreated and pressed Opal Film 2. Surface (**a**) and cross-section (**b**) of untreated opal film. Surface (**c**) and cross-section (**d**) of the pressed opal film.

Due to the significant cross-linked sites and therefore fixed state of the opal film structure, it was possible to record SEM images of the untreated and the compressed film. Figure 13a,c show images of the opal film topography. From these SEM images, it can be concluded that the untreated film (Figure 13a) still featured a much higher order of the underlying core particles at the film surface. The compressed film (Figure 13c) also revealed more pronounced particle deformation. Although the images were carried out at the same magnification, it can be concluded that the core particles of the compressed opal film had a slightly increased diameter (280 nm for the compressed opal film and 245 nm for the untreated opal film), i.e., a slightly ellipsoidal shape. Figure 13b,d show the cross-section SEM image of the corresponding opal films. Here, the individual core particles were clearly visible within both SEM images. It is noteworthy that it was possible to observe that the particles of the compressed film (Figure 13d) featured a more ellipsoidal shape rather than a spherical shape, as shown for the untreated film (Figure 13b).

**Figure 14:**
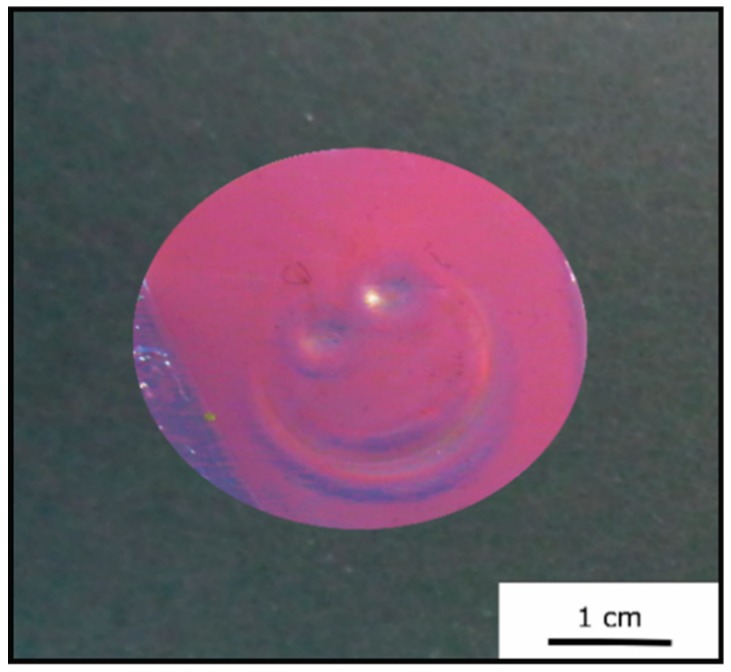
Photography of the Opal Film 2 after a pattern that was obtained followed by fixation upon UV irradiation for 5 min from each side.

This additional subsequent irradiation-mediated cross-linking step irreversibly fixed the patterned structures for the herein investigated opal films. In general, if a pattern, which featured a green reflection color in this particular case, was not satisfactory, it could be simply relaxed within 30 min. If the image was considered to be satisfactory, it can be fixed by additional irradiation of the opal film, and the pattern remained fixed (see Figure 14).

## 4. Conclusions

In conclusion, an efficient protocol for the preparation of monodisperse core-interlayer-shell particles via emulsion polymerization based on fluoropolymer-containing functional materials was described. The obtained particles were determined by TEM and DLS measurements, proving their well-defined particle architecture based on soft core-hard interlayer-soft shell particles. The tailor-made particles were subjected by means of the melt-shear organization technique for the production of photonic crystals. The resulting opal films featured brilliant reflection colors, which were investigated by angle-dependent UV-Vis spectroscopy measurements. Subsequent cross-linking by the incorporation of UV-cross-linking agents and subsequent UV-irradiation enabled gaining access to opal films that featured reversible (or irreversible) color change upon moderate pressure. The color changes were investigated by UV-Vis measurements, revealing that the original color could be obtained upon relaxation within a couple of minutes. Compared to these findings, the increase of interlayer thickness by chemical means and by using a higher content of cross-linking agent, the optical and mechanical properties significantly changed. It was shown that a higher shell thickness of the interlayer was accompanied with a prolonged relaxation. Moreover, the pressure-induced color change can be irreversibly fixed upon UV-mediated irradiation. It was shown that particle deformation either reversibly or irreversibly lead to the optical changes of the investigated opal films. In conclusion, the adjustable properties of the matrix and the underlying core-interlayer particles determined the resulting reflection color of these novel photonic crystals. The fascinating optical feature of the opal films caused by the reversible and irreversible color changing upon the application of moderate pressure is expected to pave the way to reversibly addressable pressure-sensitive deformation sensors or for anti-counterfeiting materials.

## Figures and Tables

**Table 1:** Two-layer model for fitting the poly (fluoro-acrylate) on a silicon wafer against air. Parameters are the refractive index n, absorption coefficient k, and the thickness of the layer, respectively.

Layer	Refractive Index *n*	Absorption Coefficient *k*	Thickness of Layer (nm)
air	1	0	∞
poly(fluoro-acrylate)	fit	0	fit
SiO*_x_*	1.5	0	1.5
Si	3.885	−0.02	∞

**Table 2:** Size of all investigated particle batches obtained by using TEM and dynamic light scattering (DLS) characterization, as well as standard deviations δ of all obtained particle size values.

Particles	TEM (d/nm)	DLS (d/nm)	Standard Deviation TEM (δ/nm)	Standard Deviation DLS (δ/nm)
Seed	60	71	7.8	0.3
Core	207	217	13.5	2.2
Core-interlayer 1	241	257	12.4	2.9
Core-interlayer 1-shell 1	289	305	20.9	2.7
Core-interlayer 2	258	266	8.9	4.6
Core-interlayer 2-shell 2	279	295	11.3	5.7
